# Subclinical Myocardial Injury in Patients Recovered from COVID-19 Pneumonia: Predictors and Longitudinal Assessment

**DOI:** 10.3390/jcdd10040179

**Published:** 2023-04-19

**Authors:** Antonella Cecchetto, Gianpaolo Torreggiani, Gabriella Guarnieri, Andrea Vianello, Giulia Baroni, Chiara Palermo, Leonardo Bertagna De Marchi, Giulia Lorenzoni, Patrizia Bartolotta, Emanuele Bertaglia, Filippo Donato, Patrizia Aruta, Sabino Iliceto, Donato Mele

**Affiliations:** 1 Department of Cardiac Thoracic Vascular Sciences and Public Health, University of Padua, 35128 Padua, Italy; 2 Respiratory Pathophysiology Division, University of Padua, 35128 Padua, Italy

**Keywords:** post-acute COVID-19 syndrome, cardiac involvement, LVGLS, RVGLS, follow-up

## Abstract

(1) Background: Emerging data regarding patients recovered from COVID-19 are reported in the literature, but cardiac sequelae have not yet been clarified. To quickly detect any cardiac involvement at follow-up, the aims of the research were to identify: elements at admission predisposing subclinical myocardial injury at follow up; the relationship between subclinical myocardial injury and multiparametric evaluation at follow-up; and subclinical myocardial injury longitudinal evolution. (2) Methods and Results: A total of 229 consecutive patients hospitalised for moderate to severe COVID-19 pneumonia were initially enrolled, of which 225 were available for follow-up. All patients underwent a first follow-up visit, which included a clinical evaluation, a laboratory test, echocardiography, a six-minute walking test (6MWT), and a pulmonary functional test. Of the 225 patients, 43 (19%) underwent a second follow-up visit. The median time to the first follow-up after discharge was 5 months, and the median time to the second follow-up after discharge was 12 months. Left ventricular global longitudinal strain (LVGLS) and right ventricular free wall strain (RVFWS) were reduced in 36% (n = 81) and 7.2% (n = 16) of the patients, respectively, at first the follow-up visit. LVGLS impairment showed correlations with patients of male gender (*p* 0.008, OR 2.32 (95% CI 1.24–4.42)), the presence of at least one cardiovascular risk factor (*p* < 0.001, OR 6.44 (95% CI 3.07–14.9)), and final oxygen saturation (*p* 0.002, OR 0.99 (95% CI 0.98–1)) for the 6MWTs. Subclinical myocardial dysfunction had not significantly improved at the 12-month follow-ups. (3) Conclusions: in patients recovered from COVID-19 pneumonia, left ventricular subclinical myocardial injury was related to cardiovascular risk factors and appeared stable during follow-up.

## 1. Introduction

COVID-19 infection caused by SARS-CoV-2 has been exhibiting a high morbidity and mortality secondary to the COVID-19 pandemic worldwide. In addition to the respiratory system, the cardiovascular system is also involved [1,2]. Several mechanisms could cause acute myocardial injury [3,4], such as direct cardiac damage [5], hypoxia-induced myocardial damage [6], cytokine storms [6], and macro- and micro-circulatory thrombosis [7]. Furthermore, patients with pre-existing cardiovascular disease and/or cardiovascular risk factors are more susceptible to severe complications and higher mortality rates from COVID-19 disease [8]. During its acute phase, various forms of myocardial injury have been reported using cardiac troponin levels, transthoracic echocardiography, and cardiac magnetic resonance imaging [9,10,11,12,13,14]. Two-dimensional speckle tracking echocardiography (2D-STE) can diagnose subclinical myocardial dysfunction earlier than conventional echocardiography by estimating the longitudinal strain (LS). Various studies have reported a correlation between reduced LVGLS, troponin and C-reactive protein (CRP) levels, and severity of pneumonia during hospitalisation for COVID-19 infection [15,16,17,18]; however, these correlations were not confirmed in other studies [19,20]. The lungs are the major organ involved in COVID-19, and so the RV can be affected because of increased RV afterload. RV subclinical dysfunction has been associated with worse prognoses in hospitalised patients with COVID-19, and it is correlated with mortality more than the conventional parameters of RV function. RV involvement has also been associated with increased D-dimer and CRP levels [21]. 

There are emerging data about cardiac involvement in patients recovered from COVID-19 infection. However, the causes and evolution of subclinical damage in patients recovered from COVID-19 are not yet clear. Moreover, the late phase of COVID-19, now identified by the acronym PASC (post-acute sequelae of SARS-CoV-2 infection), includes pneumological and cardiological symptoms [22] whose pathogenesis has not yet been elucidated. There is an agreement that these patients require a multidisciplinary approach with a careful symptom evaluation by functional examination [23]. While data on pulmonary function are present in the literature, less data are available regarding cardiac function in patients with PASC. 

Therefore, the aims of this study were to identify: (I) clinical and laboratory tests of the acute phase predisposing LS impairment at follow-up visits; (II) the relationship between LS impairment and clinical, laboratory, and functional parameters at follow-up visits; and (III) the longitudinal evolution of LS impairment.

## 2. Materials and Methods

### 2.1. Study Design and Population

**Figure 1:**
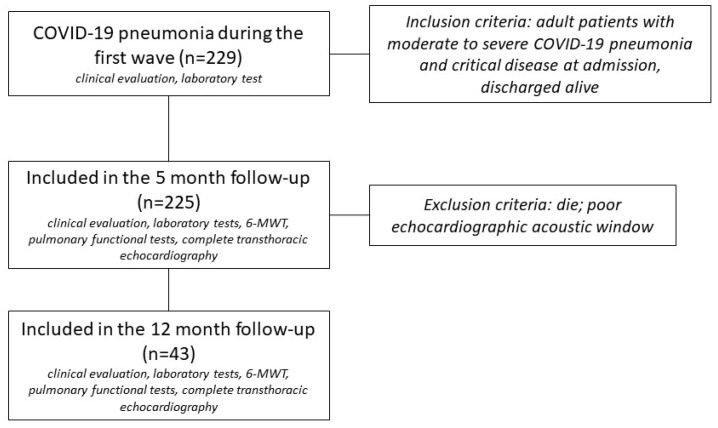
Study design.

This was a single-centre cohort study. The study protocol was approved by the Ethics Committee on Human Research (no. 20009). The investigation conformed to the principles outlined in the Declaration of Helsinki [24]. According to the fifth edition of the diagnosis and treatment plan issued by the National Health Commission, the clinical classification of COVID-19 infection should meet the following criteria: (1) mild: mild clinical symptoms and no signs of pneumonia on imaging; (2) moderate: fever and respiratory symptoms, etc. with pneumonia signs on imaging; (3) severe: patients with any of the following conditions: respiratory distress with a respiratory rate of >30 breaths/min, a peripheral capillary oxygen saturation (SpO2) of <93% at rest; a ratio of arterial oxygen partial pressure (PAO2) to fractional inspired oxygen (FiO2), or PAO2/FiO2, of <300 mmHg; and (4) critically ill: patients with respiratory failure requiring mechanical ventilation, shock, or other organ failure requiring admission to the intensive care unit. We enrolled 229 consecutive patients older than 18 years with moderate to severe COVID-19 pneumonia and critical disease at admission who had been admitted to intensive and sub-intensive unit care and were hospitalised from 18 February 2020 to 10 November 2021 and discharged alive. COVID-19 diagnosis at admission was accepted as the positive polymerase chain reaction test of the nasopharyngeal swab. Patients were considered as recovered when discharged from hospital and with a negative swab test. We excluded 3 patients for poor echocardiographic acoustic windows and 1 patient who died before the follow-up visit. Follow-up at 5 months was performed for all patients (n = 225). A second follow-up was undertaken by 19% (n = 43) of patients. The study design is shown in Figure 1. Baseline biomarkers (troponin, B-type natriuretic peptide (BNP), CPR, and D-dimer) and clinical characteristics (demographic data, medication history, coexisting medical conditions, smoking history, body mass index (BMI), type of ventilation, and duration of hospitalisation) were retrospectively collected from medical records. At the follow-up visits, all patients underwent a detailed clinical evaluation, laboratory tests (troponin, BNP, CPR, and D-dimer), a six-minute walking test (6-MWT), a pulmonary functional test, and complete transthoracic echocardiography. Specifically, the biventricular LS was calculated for each patient by 2D-STE as an index of subclinical myocardial injury. Clinical examinations assessed the persistence of cardiopulmonary symptoms such as dyspnoea using the mMRC dyspnoea scale range of 0–4 [25]. 

### 2.2. Transthoracic Echocardiography

For the transthoracic echocardiography, all acquisitions were performed using Vivid E9 (GE Vingmed, Horten, Norway) equipped with M5S and a 4V matrix probe by one experienced researcher blinded to the clinical and laboratory data. The echocardiogram was performed on the same day as the clinical and pulmonary evaluation. Data sets were digitally stored and exported on a dedicated workstation for subsequent analysis using EchoPac BT 13 (GE Vingmed, Horten, Norway). We performed comprehensive assessments of the biventricular and atrial size, left and right ventricular diastolic function, and systolic function using two-dimensional (2D) and three-dimensional (3D) technology and 2D-STE for the LS measurements in accordance with the guidelines of the EACVI (European Association of Cardiovascular Imaging) and ASE (American Society of Echocardiography) [26]. In particular, the echo parameters reported were: the LV end-systolic volume (LVESV), LV end-diastolic volume (LVEDV), and LV ejection fraction (LVEF), which were measured using the biplane Simpson method and 3D echo; the LV mass calculated on the basis of Devereux’s formula; the LV diastolic function estimated using the ratio of early transmitral flow velocity (E) to late transmitral flow velocity (A) and the ratio of transmitral E to early diastolic medial septal and lateral tissue velocity (e’); the left atrial volume calculated using the biplane method in four- and two-chamber views; the right atrial volume and area from the apical 4-chamber view; the RV end-diastolic area (RVEDA) and RV end-systolic area (RVESA) from the apical 4-chamber view; the RV fractional area change (RVFAC); the RV end-systolic volume (RVESV), RV end-diastolic volume (RVEDV), and RV ejection fraction (RVEF), which were measured using 3D echo; the RV basal diameter; the RV outflow parasternal short axis distal diameter; the RV subcostal wall thickness; the tricuspid annular plane systolic excursion (TAPSE) measured as the systolic displacement of the tricuspid lateral annulus on M-mode imaging; the tricuspid lateral annular systolic velocity (TDI S’) assessed using tissue Doppler imaging from the apical 4-chamber view; the RV diastolic function estimated using the ratio of transtricuspid E and A; the pulmonary artery systolic pressure (sPAP) assessed from the peak velocity of the tricuspid regurgitation jet using the modified Bernoulli equation plus the right atrial pressure evaluated from the inferior vena cava size and its collapsibility; the eccentricity index of the LV; the myocardial performance index (MPI) by tissue Doppler echocardiography; the early and end-diastolic pulmonary regurgitation velocities; the pulmonary artery (PA) acceleration time (AT); and the PA diameter. In addition, the probability of pulmonary hypertension was estimated in accordance with the international guidelines [27]. Two-dimensional STE was utilised to characterise the LS. Images were acquired at 70–100 frames per second from apical views and analysed in a blinded manner offline using a dedicated software package (Automatic Function Imaging (AFI), EchoPac.PC; GE Healthcare, Chicago, IL, USA). Using the AFI, a point-and-click approach was utilised to identify three anchor points (two basal and one apical), following which the software tracked the endocardial border of the LV and RV automatically. Manual adjustment was performed to ensure adequate tracking. The LS was obtained by comparing the displacement of the speckles relative to each other throughout the cardiac cycle for each segment. The LV was divided into 17 segments for analysis, and we reported the average value or LVGLS. The RV was divided into 6 segments for analysis (basal free wall, mid-free wall, apical free wall, basal septum, mid septum, and apical septum). We considered the RVFWS calculated as the average of the strain values in the 3 segments of the RV free wall. The standard LVGLS limit was identified as −18% and the RVFWS was identified as −20% [26]. 

### 2.3. Pulmonary Functional Test

Pulmonary functional tests and lung diffusing capacity for carbon monoxide (DLCO) measurements were performed by an experienced researcher using calibrated equipment (MasterLab Pro; Erich Jaeger GmbH; Höchberg, Germany) according to the European Respiratory Society (ERS) and American Thoracic Society (ATS) recommendations [28,29]. The predicted normal values of Quanjer [30] and equations of Cotes for DLCO [31] were used. Among the various parameters of the spirometry, vital capacity (VC), total lung capacity (TLC), lung diffusion capacity for carbon monoxide (DLCO), carbon monoxide transfer coefficient (KCO), maximal inspiratory pressure (MIP), maximal expiratory pressure (MEP), and Tiffeneau index were included in the analysis. For each patient, the parameters were also expressed as a percentage of a theoretical value. 

### 2.4. Six-Minute Walking Test

A 6MWT was performed according to recommended guidelines [32] with baseline and after-exercise SpO2 measurements by pulse oximetry on index fingers. Desaturation was defined as a drop of ≥4% and a reduction of <90% in SpO2. Self-reported intensities of exertion on the Borg rating of perceived exertion were collected before and after exercise. Walking capacity was considered abnormal when it was below the cut-off value of a similar cohort of healthy patients (484 mt). 

### 2.5. Statistical Analysis

Data were reported as medians and interquartile ranges (IQRs, I–III quartile) for continuous variables and as absolute values and percentages for categorical variables. Univariable and multivariable logistic regression analyses were used to estimate the effects of different variables on the presence of LS (LVGLS and RVFWS) impairment at follow-up visits. Multivariable model selection was performed according to the Bayesian Information Criterion (BIC). The results were reported as odds ratios (ORs) with 95% confidence intervals (CIs). The characteristics of patients at two follow-up visits were tested for significant differences with Wilcoxon rank sum tests and Chi squared tests based on the types of variables taken into consideration. Statistical significance was taken as *p* < 0.05. Statistical analysis was performed using R software version 4.1.0 222.

## 3. Results

### 3.1. Characteristics at Hospitalisation 

Our study group included patients recovered from the first wave of COVID-19. The patients’ characteristics are displayed in Table 1. 

A total of 229 consecutive patients were initially enrolled, of which 225 were available for follow-up. The patients presented with a median age of 65 years (IQR 55–73) and a median duration of hospitalisation of 18 days (IQR 12–28). The demographical information highlighted that our patients were mainly overweight adults (median BMI of 28.1 kg/m^2^ (IQR 24–31)) without histories of CVD (n = 213, 95%) but with several cardiovascular comorbidities (n = 151, 67%) and histories of smoking (5%, n = 11 smoker and 39%, n = 88 former smoker). Most of them (n = 188, 84%) had received oxygen therapy. Of our patients, troponin increases above the reference values were presented by 16% (n = 36), and BNP increases were presented by 64% (n = 144) during hospitalisation. All patients manifested signs of infection (median CRP of 120 mg/L (IQR 81–180)). 

### 3.2. Characteristics at First Follow-Up Visit

The characteristics of the patients at the first follow-up visits are displayed in Table 2 and Table 3. The median time to the first follow-up after discharge was 5 months (IQR 3.8–7.5). At follow-up, approximately half of the sample (n = 115, 51%) reported dyspnoea. Concerning laboratory blood tests, CRP values had returned to normal ranges in 84% (n = 188) of the patients, proving recovery from the infectious disease. We observed a normalisation of troponin and BNP values in 99% (n = 223) and 97% (n = 218) of patients, respectively. At 6MWT, we observed a reduction in the total walking distance covered in 41% (n = 92) of patients and a desaturation in 32% (n = 72) of patients. Indeed, regarding the respiratory data from the pulmonary functional tests, the sample was characterised by normal pulmonary volumes without signs of lung restrictive patterns but with tendencies toward reductions in (i) lung diffusion capacities (median DLCO 76%, IQR (61–87)) and (ii) respiratory muscle strength during both inhalation and exhalation (median MIP 74 cmH_2_O, (IQR 56–103) and median MEP 90 cmH_2_O, IQR (68–113), respectively). Concerning the echocardiographic data, the sample showed a trend toward normal cardiac structures (i.e., volumes and systolic and diastolic functions), and in particular, there were 3D LVEDV increase in 10% of the patients, 3D LVEF reductions in 9% of the patients, LV diastolic disfunction in 12% of the patients, 3D RVEDV increases in 28% of the patients, and 3D RVEF reductions in 8% of the patients. Only 2% of the cohort demonstrated an intermediate to high probability of pulmonary hypertension, which was evaluated according to international guidelines [25]. Considering the first follow-up, 81 patients (36%) had LVGLS reductions (>−18%) and 16 patients (7.2%) had RVFWS reductions (>−20%). 

### 3.3. Predictors of LV Subclinical Impairment

The results of the logistic regression analysis that aimed to identify the predictors of LV subclinical dysfunction among the clinical and laboratory variables obtained during the hospitalisation are presented in Table 4. The significant predictors of LVGLS impairment included the presence of at least one cardiovascular risk factor (*p* < 0.001, OR 6.44 (95% CI 3.07–14.9)) and being a patient of male gender (*p* 0.008, OR 2.32 (95% CI 1.24–4.42)). The logistic regression analysis aimed to correlate the variables obtained during the 5-month visit with LV subclinical dysfunction, and it showed that the final SpO2 at the 6MWT (*p* 0.002, OR 0.99 (95% CI 0.98–1.00)) was a significant predictor (Table 5). 

### 3.4. Predictors of RV Subclinical Impairment

No significant predictors of hospitalisation and follow-up were found for RV subclinical dysfunction. 

### 3.5. Longitudinal Evolution of Follow-Up Parameters 

A total of 43 patients (19%) attended second follow-up visits. The median time to the second follow-up after discharge was 12 months. We evaluated the longitudinal trends of: (i) the pulmonary functional test data; (ii) the exercise tolerance by the 6MWT; and (iii) the echocardiographic data. The results are displayed in Table 6, and we determined the presence of a significant improvement in the respiratory parameters (e.g., volume and lung diffusivity). We did not see any significant LVGLS and RVFWS variations. 

## 4. Discussion

The present study is the first to investigate recovery following moderate to severe COVID-19 pneumonia using full echocardiographic examinations, pulmonary functional tests, and 6MWTs, along with cardiac biomarker sampling performed during both the acute course of the infection and the recovery phase at 5 months. Furthermore, for the first time, cardiopulmonary data and exercise tolerance were evaluated longitudinally through a second follow-up visit at 12 months. We found that: (i) LVGLS and RVFWS were respectively reduced in 36% and 7.2% of patients after 5 months from hospitalisation; (ii) LVGLS and RVFWS did not manifest correlations with the acute disease parameters (CRP, troponin, BNP, days of hospitalisation, and type of ventilation), and only LVGLS was correlated with the presence of at least one cardiovascular risk factor; (iii) considering the follow-up parameters, the final SpO2 at the 6MWT predicted the LVGLS impairment; and (iv) the echocardiographic parameters of subclinical LV and RV dysfunction did not improve significantly at the 12-month follow-up. 

We used 2D-STE, in addition to the standard 2D and 3D echocardiographic assessments, in order to evaluate the long-term subclinical effects of COVID-19 on LV and RV function. In this study, we confirmed reductions in LVGLS (>−18%) and RVFWS (>−20%), respectively, in 36% and 7.2% of the patients after 5 months from hospitalisation for COVID-19 pneumonia. Regarding the LV, our results are in line with those reported in the literature, where Ozer et al. [33] and Mahajan et al. [34] described a deterioration in LVGLS values in 37.8% and 29.9% of all patients one month after discharge. However, the clinical conditions that may cause deterioration in LVGLS analysis were determined as the exclusion criteria. In the same way, Shimoni et al. [35] and Raafs et al. [36] confirmed, respectively, that 2 months after recovery, there was an LVGLS impairment in 37% of the sample (patients with known cardiac disease were excluded), and 6 months after recovery, there was an LVGLS impairment in 24% of the sample. Despite the results in the literature, RV subclinical dysfunction is a common finding, and in our population, only 7.2% of the patients presented with a reduction in RVFWS. We decided to exclude the septum from the measurement in order to have more specific data for RV function. Rameshwar et al. [37] reported a subclinical RV dysfunction in 21% of patients, but they considered an RVGLS impairment with different cut-off value (>−24%) and they performed the study 2 weeks after discharge. 

We demonstrated the absence of a correlation between LV and RV subclinical dysfunction at follow-up and clinical and laboratory parameters of acute disease severity (CRP, troponin, D-dimer, BNP, days of hospitalisation, and pulmonary compromise in relation to the type of ventilatory support); however, the recently published data do not always agree, likely due to different patient selection criteria and different follow-up lengths. van den Heuvel et al. [38] confirmed that elevated troponin and/or NT-proBNP levels during hospitalisation were not associated with myocardial function at 4-month follow-up visits. Subjects with reduced LVGLS levels at the one-month follow-up had significantly higher CPR and troponin levels during index admission in the study by Mahajan et al. [34]. Ozer et al. [33] divided their study population into two groups according to the increase, or lack thereof, in troponin levels, finding higher LV subclinical dysfunction rates in the first group (57.1%) compared to the second group (26.1%). Therefore, in this study, which was performed only one month after healing, the reductions in LVGLS were not excluded in patients with normal troponin values. Furthermore, causes that may have increased troponin levels or clinical conditions that may have caused deteriorations in LVGLS analyses were determined to be exclusion criteria, but cardiovascular risk factors, such as hypertension and diabetes, which are present in more than 50% of the population, were not identified as exclusion criteria. Regarding our results, we could not rule out that in the acute phase, as demonstrated in the literature, there may be a relationship between COVID-19 infection and LV subclinical damage, but this damage may not necessarily remain significant after healing. Bieber et al., for example, described the partial resolution of LV dysfunction with 2D-STE at the 2-month follow-up [39]. In support of this hypothesis, we found a correlation between the LVGLS reduction observed at the 5-month follow-up and the presence of cardiovascular risk factors; thus, it could be a condition that existed prior to COVID-19 infection. Considering the relationship between cardiovascular risk factors and subclinical myocardial damage assessed by 2D-STE, which has been demonstrated in the literature [40], it could be independent of COVID-19. Therefore, severe COVID-19 infection may not be responsible by itself for subclinical myocardial damage and may not warrant long-term echocardiographic follow-up.

Regarding RV subclinical dysfunction at follow-up, some studies have demonstrated its relationship with pneumonia severity [21] and others have demonstrated the persistence of RVLS reductions in patients with mild pneumonia [41]. The possible mechanism of RV dilation and dysfunction is multifactorial. Thrombotic events, hypoxic vasoconstriction, direct viral damage, proinflammatory cytokines, and, most likely, increased afterload and overload are some of these mechanisms. Therefore, acute COVID-19 negatively affected RV function, but it was recovered following the resolution of COVID-19 [39,40,41,42]. 

Considering the follow-up parameters, we did not find a correlation between LVGLS and RVFWS reductions and pulmonary functional parameters. Instead, the final SpO2 at the 6MWT was able to predict LVGLS impairment. This was likely because patients with subclinical LV dysfunction (who presented a correlation with cardiovascular risk factors) were also the most deconditioned from a physical point of view. In the literature, while 45% of COVID-19 patients showed a performance of below the lower limit of normal at the 6MWT (6 weeks after infection), more than 20% of the patients would still have had such limitations 6 months thereafter regardless of the severity of the acute illness [43,44]. Although the pathogenesis of these limitations is likely multifactorial (muscular, cardiopulmonary, psychological, etc.), cardiopulmonary evaluation is always required in patients with impaired exercise capacity. However, data about the cardiac function in patients after COVID-19 infection is sparse and continues to evolve.

Finally, in our study, for the first time, we performed two consequential follow-up visits for complete cardiovascular and pulmonary evaluation. We identified an improvement in pulmonary function, and the LVGLS and RVFWS were stable at the 12-month follow up, emphasizing the possibility that subclinical alterations of the LV may exist prior to COVID-19 infection and could be related to a patient’s cardiovascular risk factors.

Some limitations of this single-centre study should be highlighted. Our study did not include multi-parametric evaluations, even in the acute phase of hospitalisation, due to the clinical restrictions posed by COVID-19 infections. We included only hospitalised patients who had been admitted to intensive and sub-intensive unit care and successfully recovered from COVID-19; therefore, we disregarded patients who died during hospitalisation. Patients admitted to medical departments were also excluded. An echocardiogram was not available before the infection, and lastly, our longitudinal evaluation could be further improved by extending the follow-up period to a higher number of patients.

## 5. Conclusions

Biventricular subclinical myocardial injuries found during follow-up visits with patients recovered from COVID-19 pneumonia did not manifest a correlation with acute disease parameters, but they were only related to cardiovascular risk factors and were stable at subsequent echocardiographic controls. Long-term studies are needed to better understand the cardiac limitations after COVID-19 and to assess their clinical implications and progression over time.

## Figures and Tables

**Table 1:** Demographic and clinical characteristics at hospitalisation.

Age (y) (median IQR [range])	65 [55–73]
Gender (F (%), M (%))	88 (39%), 137 (61%)
Smoke (n (%))	No (125 (56%)) User (11 (5%)) Former smoker (88 (39%))
Body mass index (kg/m^2^) (median IQR [range])	28.1 [24–31.4]
Previous cardiovascular disease (n (%))	Absent (213 (95%)) Present (12 (5.3%))
Type of cardiovascular disease (n (%))	Ischemic disease (8 (3.5%)) Valvular disease (3 (1.3%)) HCM (1 (0.4%))
Duration of hospitalisation (days) (median IQR [range])	18 [12, 28]
Respiratory ventilation (type (n (%)))	Absent (36 (16%)) NC-SM (25 (11%)) RM (6 (3%)) HFNC (69 (31%)) NIV (28 (13%)) MV (54 (25%)) ECMO (2 (1%))
Troponin I (ng/L) (median IQR [range]) (n.v. < 34 ng/L)	9 [5, 26]
CRP (mg/L) (median IQR [range]) (n.v. < 5 mg/L)	120 [81, 180]
D-dimer (ng/mL) (median IQR [range]) (n.v. < 500 ng/mL)	662 [295, 1927]
BNP (pg/mL) (median IQR [range]) (n.v. < 125 pg/mL)	70 [26, 120]
Cardiovascular risk factors (n (%))	Absent (74 (33%)) Present (151 (67%))
Cardiovascular risk factors type (n (%))	Hypertension (101 (45%)) Dyslipidaemia (33 (14%)) Diabetes Mellitus (34 (15%)) Obesity (58 (26%)) CKD (7 (3%))

Note: n = 225; interquartile range (IQR); nasal cannula (NC); simple face mask (SM); non-rebreather mask (RM); high-flow nasal cannula (HFNC); non-invasive ventilation (NIV); mechanical ventilation (MV); extracorporeal membrane oxygenation (ECMO); C-reactive protein (CRP); B-type natriuretic peptide (BNP); chronic kidney disease (CKD); normal value (n.v.).

**Table 2:** Characteristics at the first follow-up visits (clinical and instrumental evaluations), with differences between the patients with and without subclinical injuries.

	Total Patients (n = 225)	Patients with LVGLS of >−18% (n = 81)	Patients with LVGLS of ≤−18% (n = 144)	Patients with RVFWS of >−20% (n = 16)	Patients with RVFWS of ≤−20% (n = 209)
Days from hospitalisation (median IQR, [range])	154 [113, 225]	130 [115, 212]	149 [107, 228]	122 [84, 212]	144 [112, 222]
Presence of dyspnoea at follow-up (n (%))	Yes (115 (51%)) No (110 (49%))	Yes (43 (53%)) No (38 (47%))	Yes (73 (51%)) No (71 (49%))	Yes (10 (63%)) No (6 (37%))	Yes (106 (51%)) No (103 (49%))
Grade of dyspnoea (mMRC scale) (n (%))	0 (43 (19%)) 1 (38 (17%)) 2 (22 (10%)) 3 (12 (5%))	0 (17 (21%)) 1 (11 (14%)) 2 (8 (10%)) 3 (7 (9%))	0 (29 (20%)) 1 (21 (15%)) 2 (14 (10%)) 3 (9 (6%))	0 (2 (13%)) 1 (2 (13%)) 2 (3 (18%)) 3 (3 (18%))	0 (45 (22%)) 1 (27 (13%)) 2 (27 (13%)) 3 (7 (3%))
Laboratory tests					
Troponin (ng/L) (median IQR [range])	2 [2.0, 4.0]	2.6 [2.0, 5.7]	2 [2.0, 2.7]	2.1 [2, 7.2]	2 [2, 3]
CRP (mg/L) (median IQR [range])	2.9 [2.9, 3.0]	2.9 [2.9, 4.1]	2.9 [2.9, 2.9]	2.9 [2.9, 3.6]	2.9 [2.9, 2.9]
D-dimer (ng/L) (median IQR [range])	150 [150, 154]	150 [150, 184]	150 [150, 150]	150 [150, 228]	150 [150, 150]
BNP (pg/L) (median IQR [range])	22 [12, 46]	130 [115, 212]	22 [13, 43]	30 [11, 50]	22 [13, 45]
Six-minute walking test					
Distance (m) (median IQR [range])	420 [360, 480]	420 [360, 450]	450 [375, 495]	390 [300, 450]	450 [368, 480]
Final SpO2 (%) (median IQR [range])	96 [95, 98]	96 [94, 97]	97 [95, 98]	96 [91, 97]	97 [95, 98]
Absolute drop in SpO2 (%) (median IQR [range])	2 [1, 3]	2 [1, 4]	2 [1, 4]	2 [2, 3]	2 [1, 3.5]
Pulmonary functional test					
VC (L) (median IQR [range])	3.6 [2.9, 4.2]	3.5 [2.8, 4.1]	3.6 [2.9, 4.2]	3.5 [2.7, 4.2]	3.6 [2.9, 4.3]
VC (%) (median IQR [range])	102 [91, 114]	96 [84, 110]	105 [95, 116]	87 [84, 91]	104 [92, 114]
TLC (L) (median IQR [range])	5.6 [4.6, 6.6]	5.8 [4.7, 6.6]	5.6 [4.5, 6.6]	5.6 [4.4, 6.3]	5.8 [4.7, 6.7]
TLC (%) (median IQR [range])	95 [86, 103]	92 [82, 102]	96 [88, 105]	85 [73, 94]	95 [87, 105]
DLCO (mL/min/mmHg) median IQR [range])	19 [14, 23]	19 [14, 23]	19 [14, 23]	20 [12, 23]	20 [14, 24]
DLCO (%) (median IQR [range])	76 [61, 87]	75 [62, 87]	79 [61, 88]	68 [56, 78]	78 [62, 90]
KCO (L) (median IQR [range])	3.6 [2.9, 4.1]	3.6 [2.8, 4.2]	3.6 [3, 4]	3.8 [3, 4]	3.65 [3, 4]
MIP (cmH_2_O) (median IQR [range])	74 [56, 103]	80 [57, 98]	73 [58, 104]	94 [41, 126]	78 [59, 104]
MEP (cmH_2_O) (median IQR [range])	90 [68, 113]	92 [74, 118]	90 [68, 110]	86 [64, 127]	90 [70, 113]
Tiffeneau index (%) (median IQR [range])	0.84 [0.79, 0.88]	0.85 [0.79, 0.89]	0.84 [0.8, 0.87]	0.87 [0.81, 0.90]	0.84 [0.79, 0.88]

Note: interquartile range (IQR); C-reactive protein (CRP); B-type natriuretic peptide (BNP); peripheral capillary oxygen saturation (SpO2); vital capacity (VC); total lung capacity (TLC); lung diffusion capacity for carbon monoxide (DLCO); carbon monoxide transfer coefficient (KCO); maximum inspiratory pressure (MIP); maximum expiratory pressure (MEP).

**Table 3:** Characteristics at the first follow-up visits (echocardiogram parameters) with differences between the patients with and without subclinical injuries.

Transthoracic Echocardiography					
	Total Patients (n = 225)	Patients with LVGLS of >−18% (n = 81)	Patients with LVGLS of ≤−18% (n = 144)	Patients with RVFWS of >−20% (n = 16)	Patients with RVFWS of ≤−20% (n = 209)
LVEDVi biplane (mL/m^2^) (median IQR [range])	51 [43, 58]	51 [43, 59]	51 [44, 58]	49 [41, 57]	51 [44, 58]
LVEDVi 3D (mL/m^2^) (median IQR [range])	54 [47, 62]	54 [45, 64]	55 [49, 62]	56 [46, 49]	55 [47, 53]
LVESVi biplane (mL/m^2^) (median IQR [range])	20 [16, 23]	21 [17, 25]	19 [16, 22]	19 [14, 24]	20 [16, 24]
LVESVi 3D (mL/m^2^) (median IQR [range])	21 [18, 25]	23 [19, 28]	20 [17, 23]	22 [18, 26]	21 [18, 25]
LVEF biplane (%) (median IQR [range])	61 [57, 65]	58 [55, 62]	63 [59, 66]	64 [57, 66]	61 [57, 64]
LVEF 3D (%) (median IQR [range])	61 [58, 64]	58 [55, 60]	62 [59, 65]	58 [57, 63]	60 [58, 64]
LVGLS (%) (median IQR [range])	−18.6 [−20, −17]	−16.4 [−17, −15]	−20 [−21, −19]	−17 [−19, −15]	−18.6 [0.20, −17]
E/A ratio (median IQR [range])	0.85 [0.71, 1.08]	0.77 [0.62, 0.96]	0.94 [0.78, 1.17]	0.92 [0.71, 1.08]	0.88 [0.73, 1.09]
E/E’ ratio (median IQR [range])	7.55 [6.0, 9.11]	8.11 [6.3, 9.7]	6.93 [5.8, 8.8]	8.61 [6.7, 9.9]	7 [6, 9]
Left atrial volume index (mL/m^2^) median IQR [range])	30 [25, 35]	29 [25, 36]	30 [26, 35]	28 [24, 34]	30 [26, 36]
RVEDAi (cm^2^/m^2^) (median IQR [range])	11 [9, 12]	10 [9, 11]	11 [9, 12]	10 [9, 12]	11 [10, 12]
RVESAi (cm^2^/m^2^) (median IQR [range])	6 [5, 7]	6 [5, 7]	6 [5, 7]	7 [5, 7]	6 [5, 7]
FAC (%) (median IQR [range])	44 [40, 47]	43 [40, 48]	45 [41, 47]	41 [37, 43]	44 [41, 48]
RVEDVi 3D (mL/m^2^) (median IQR [range])	50 [41, 61]	44 [38, 58]	52 [42, 61]	51 [36, 57]	50 [41, 60]
RVESVi 3D (mL/m^2^) (median IQR [range])	23 [19, 30]	23 [20, 28]	24 [19, 32]	27 [21, 31]	23 [19, 29]
RVEF 3D (%) (median IQR [range])	52 [47, 56]	52 [48, 54]	52 [47, 57]	50 [44, 53]	52 [48, 57]
RVFWS (%) (median IQR [range])	−24.4 [−27, −22]	−23 [−26, −21]	−22 [−28, −22]	−18 [−18.6, −17]	−25 [−28, −22]
TAPSE (mm) (median IQR [range])	22 [20, 24]	21 [20, 24]	22 [20, 25]	22 [19, 24]	22 [20, 24]
Probability of pulmonary hypertension (n (%))					
Low	(193 (93%))	(72 (89%))	(124 (86%))	(15 (94%))	(189 (90%))
Intermediate	(11 (5%))	(5 (6%))	(20 (14%))		(18 (9%))
Intermediate–high	(3 (1.4%))	3 (4%))		(1 (6%))	(2 (1%))
High	(1 (0.5%))	(1 (1%))			
SPAP (mmHg) (median IQR [range])	26 [21, 29]	26 [22, 32]	26 [21, 29]	27 [21, 29]	26 [22, 30]
PVR (WU) (median IQR [range])	1.73 (1.49, 1.99)	1.85 [1.61, 2.08]	1.67 [1.42, 1.96]	1.67 [1.41, 2.15]	171.4 [1.5, 1.99]

Note: interquartile range (IQR); left ventricle (LV); right ventricle (RV); end-diastolic volume index (EDVi); end-systolic volume index (ESVi); ejection fraction (EF); left ventricle global longitudinal strain (LVGLS); right ventricle end-diastolic area index (RVEDAi); right ventricle end-systolic area index (RVEDAi); fractional area change (FAC); right ventricle free-wall strain (RV FWS); right ventricle global longitudinal strain (RVGLS); tricuspid annular plane excursion (TAPSE); systolic pulmonary artery pressure (sPAP); pulmonary vascular resistance (PVR).

**Table 4:** Acute predictors of left ventricular subclinical dysfunction.

	Univariable				Multivariable	
Characteristic	OR	95% CI	*p*-Value	OR	95% CI	*p*-Value
Age	1.03	1.00, 1.05	0.033			
Gender			0.007			0.008
Male	2.24	1.25, 4.12		2.32	1.24, 4.42	
Tobacco consumption			0.053			
BMI	1.05	1.00, 1.11	0.057			
Days of hospitalisation	0.99	0.97, 1.01	0.2			
Troponin I	1.00	1.00, 1.00	0.3			
CRP	1.00	1.0, 1.00	0.5			
D-dimer	1.00	1.00, 1.00	0.3			
BNP	1.00	1.00, 1.00	0.3			
CV risk factors	5.77	2.85, 12.8	<0.001	6.44	3.07, 14.9	<0.001

Note: body mass index (BMI); C-reactive protein (CRP); B-type natriuretic peptide (BNP); cardiovascular (CV).

**Table 5:** Correlation between left ventricular subclinical dysfunction and the multi-parametric evaluations performed at the first follow-ups.

	Univariable				Multivariable	
Characteristic	OR	95% CI	*p*-Value	OR	95% CI	*p*-Value
Pulmonary functional test						
VC	0.89	0.74, 1.06	0.2			
VC %	0.99	0.99, 1.00	0.015			
TLC	0.92	0.83, 1.01	0.094			
TLC %	0.99	0.99, 1.00	0.11			
DLCO	0.98	0.95, 1.00	0.085			
DLCO %	0.99	0.99, 1.00	0.078			
KCO	1.00	0.99, 1.00	0.2			
MIP	1.0	0.99, 1.00	0.11			
MEP	1.0	0.99, 1.00	0.10			
Six-minute walking test						
Distance	1.00	1.00, 1.00	0.005			
Final saturation	0.99	0.99, 1.00	0.020	0.99	0.98, 1.00	0.002
Absolute drop in SpO2	0.88	0.76, 1.00	0.059			

Note: vital capacity (VC); total lung capacity (TLC); lung diffusion capacity for carbon monoxide (DLCO); carbon monoxide transfer coefficient (KCO); maximum inspiratory pressure (MIP); maximum expiratory pressure (MEP).

**Table 6:** Multi-parametric comparison (pulmonary functional test, 6MWT, and cardiac imaging data) between the two consecutive follow-up visits.

Variable	First FU	Second FU	*p*-Value
Pulmonary functional test			
VC (L)	3.17 (3.16, 3.18)	3.70 (3.17, 4.09)	0.13
VC (%)	98 (86, 109)	101 (90, 111)	<0.001
TLC (L)	5.69 (4.73, 6.21)	5.93 (4.90, 6.54)	0.002
TLC (%)	96 (83, 115)	94 (87, 105)	0.9
DLCO (mL/min/mmHg)	18 (12, 24)	20 (16, 25)	0.007
DLCO (%)	72 (50, 84)	78 (63, 94)	0.007
KCO (L)	68 (26, 113)	4 (3, 4)	<0.001
MIP (cm H_2_O)	44 (35, 60)	79 (66, 94)	0.13
MEP (cm H_2_O)	65 (60, 76)	91 (71, 105)	0.9
Six-minute walking test			
Distance (m)	435 (390, 480)	435 (382, 458)	0.7
Final saturation (%)	96 (95, 97)	96 (95, 96)	0.6
Echocardiographic ventricular dysfunction			
LVGLS (%)	−18.5 (−20.4, −16.8)	−18.6 (−20.6, −17.4)	0.7
RVFWS (%)	−24.3 (−27.5, −21.8)	−25.2 (−28.1, −22.0)	0.4
Left ventricular subclinical dysfunction (LVGLS of >−18%)	16 (36%)	13 (30%)	0.6
Right ventricular subclinical dysfunction (RVFWS of >−20%)	9 (22%)	4 (10%)	0.3

Note: interquartile range (IQR); FU (follow-up); vital capacity (VC); total lung capacity (TLC); lung diffusion capacity for carbon monoxide (DLCO); carbon monoxide transfer coefficient (KCO); maximum inspiratory pressure (MIP); maximum expiratory pressure (MEP); left ventricle global longitudinal strain (LVGLS); right ventricle free-wall strain (RVFWS).

## Data Availability

The data presented in this study are available on request from the corresponding author. The data are not publicly available due to privacy reason.
